# Air–Liquid Interface Exposure of Lung Epithelial Cells to Low Doses of Nanoparticles to Assess Pulmonary Adverse Effects

**DOI:** 10.3390/nano11010065

**Published:** 2020-12-29

**Authors:** Silvia Diabaté, Lucie Armand, Sivakumar Murugadoss, Marco Dilger, Susanne Fritsch-Decker, Christoph Schlager, David Béal, Marie-Edith Arnal, Mathilde Biola-Clier, Selina Ambrose, Sonja Mülhopt, Hanns-Rudolf Paur, Iseult Lynch, Eugenia Valsami-Jones, Marie Carriere, Carsten Weiss

**Affiliations:** 1 Karlsruhe Institute of Technology, Institute of Biological and Chemical Systems–Biological Information Processing, 76344 Eggenstein-Leopoldshafen, Germany; sivakumar.murugadoss@kuleuven.be (S.M.); marcodilger@gmx.de (M.D.); susanne.fritsch-decker@kit.edu (S.F.-D.); 2 CEA, CNRS, IRIG, SyMMES, University Grenoble Alpes, 38054 Grenoble, France; lucie.armand@agroparistech.fr (L.A.); david.beal@cea.fr (D.B.); marie-edith.arnal@wanadoo.fr (M.-E.A.); mathilde.clier@gmail.com (M.B.-C.); marie.carriere@cea.fr (M.C.); 3 Karlsruhe Institute of Technology, Institute for Technical Chemistry, 76344 Eggenstein-Leopoldshafen, Germany; c.schlager@vitrocell.com (C.S.); sonja.muelhopt@kit.edu (S.M.); hanns@dr-paur.net (H.-R.P.); 4 Promethean Particles Ltd., Nottingham NG7 3EF, UK; selina.ambrose@proparticles.co.uk; 5 School of Geography Earth & Environmental Sciences (GEES), University of Birmingham (UoB), Edgbaston, Birmingham B15 2TT, UK; i.lynch@bham.ac.uk (I.L.); E.ValsamiJones@bham.ac.uk (E.V.-J.)

**Keywords:** cerium dioxide, zirconium-doping, titanium dioxide, nanotoxicology, alternative methods

## Abstract

Reliable and predictive in vitro assays for hazard assessments of manufactured nanomaterials (MNMs) are still limited. Specifically, exposure systems which more realistically recapitulate the physiological conditions in the lung are needed to predict pulmonary toxicity. To this end, air-liquid interface (ALI) systems have been developed in recent years which might be better suited than conventional submerged exposure assays. However, there is still a need for rigorous side-by-side comparisons of the results obtained with the two different exposure methods considering numerous parameters, such as different MNMs, cell culture models and read outs. In this study, human A549 lung epithelial cells and differentiated THP-1 macrophages were exposed under submerged conditions to two abundant types of MNMs i.e., ceria and titania nanoparticles (NPs). Membrane integrity, metabolic activity as well as pro-inflammatory responses were recorded. For comparison, A549 monocultures were also exposed at the ALI to the same MNMs. In the case of titania NPs, genotoxicity was also investigated. In general, cells were more sensitive at the ALI compared to under classical submerged conditions. Whereas ceria NPs triggered only moderate effects, titania NPs clearly initiated cytotoxicity, pro-inflammatory gene expression and genotoxicity. Interestingly, low doses of NPs deposited at the ALI were sufficient to drive adverse outcomes, as also documented in rodent experiments. Therefore, further development of ALI systems seems promising to refine, reduce or even replace acute pulmonary toxicity studies in animals.

## 1. Introduction

Elucidation of the mechanisms responsible for the adverse effects of manufactured nanomaterials (MNMs) is necessary for the safe development and implementation of nanotechnology [1,2]. Since inhalation is a major uptake route of MNMs, a novel exposure method for pulmonary toxicity studies, the so-called interface (ALI) method, was developed in recent years [3,4]. In vitro exposure to airborne MNMs is technically challenging and labor extensive because an aerosol has to be generated, conditioned for temperature and humidity and applied to a cell surface which is not covered with medium. As submerged exposure to particle suspensions is much easier, most in vitro studies are performed this way. However, this approach does not represent the conditions which occur during inhalation [5,6], and thus, may not provide accurate hazard assessments. The particle properties will be changed by dispersion in cell culture medium, which contains a large number of biomolecules, including serum proteins. Proteins adsorb to the particles, forming a corona which may prevent adverse effects to the cells [7,8]. For submerged exposure, it is difficult to determine the particle dose correctly, because the agglomeration state is mostly unknown and the settling velocity is not defined. Furthermore, the particles may also dissolve partially in the culture medium [9] and the particle dose is often delivered as a bolus. During inhalation of aerosols, by contrast, the particles are deposited linearly over a defined period. This may have an effect on the quality and intensity of the biological effects. At KIT, the “Karlsruher Exposure System” was developed and several techniques for the validation of the exposure stations, as well as aerosol generation and cell handling, were established [10,11,12,13]. KIT, together with VITROCELL SYSTEMS, set up a first Automated Exposure Station, which has been used for the assessment of nanoscale particle emissions from combustion sources such as ship diesel and wood burners [14,15,16]. The system was further developed and offers a compact solution for toxicity testing of nanoparticle (NP) aerosols including sample conditioning, reproducible deposition, integrated dose determination by a quartz crystal microbalance (QCM), flow control, automated processes and data acquisition. The device was also tested with partner laboratories with the aim of potentially standardizing and achieving regulatory acceptance of the method.

Nevertheless, a comprehensive and rigorous comparison of results obtained from ALI or submerged exposure of cells is still lacking, and has only been performed for a few MNMs so far. Furthermore, the predictability of adverse outcomes documented in vitro given the outcome in vivo still needs to be established. Here, we compare results from submerged and ALI exposure of human lung cells to CeO_2_ and TiO_2_ NPs with particular focus on the influence of their redox potential. Both of these NPs are produced in large quantities worldwide for a variety of applications [17], and in vivo datasets for these NPs are available in the literature to benchmark the ALI results against. CeO_2_ MNMs are used, e.g., as electrodes in fuel cell technology, as catalysts in the exhaust gas treatment of cars to reduce exhaust emissions, or as a polishing agent in the semiconductor industry. TiO_2_ MNMs are used, e.g., as sun-blocker in sunscreens, in wall paints and in cosmetic products [18].

There are clear associations between the generation of reactive oxygen species (ROS) and the toxicity of MNMs [19,20]. The redox-active CeO_2_ can cycle between two redox states, Ce^3+^ and Ce^4+^, which endows this MNM with catalytic properties. Since the hypothesis underpinning the present study was that the redox potential is driving the possible toxicity of NPs based on oxidative stress, we applied chemical doping (intentional substitution of one element by another while maintaining the lattice structure and arrangement) of CeO_2_ NPs with zirconium to specifically investigate the influence of redox activity on biological effects. By doping CeO_2_ MNMs with another material, it was expected that the redox activity on the surface of the NPs would change according to the Ce(III)/Ce(IV) ratio. So, CeO_2_ MNMs with more ZrO_2_-doping were expected to have a lower Ce(III)/Ce(IV) ratio, less redox activity, less oxidative properties and, therefore, to induce less toxicity. Besides the lower redox activity, the altered chemical composition might also influence the NP toxicity. However, the influence of changes in chemical composition is expected to be limited, since the toxicities of CeO_2_ and ZrO_2_ MNMs have been shown to be similar in several in vitro and in vivo assays [21].

CeO_2_ and Zr-doped MNMs were used to study the responses in mice after inhalation [22]. Exposure to 4 mg/m^3^ for 3 h per day, 5 days/week over a four-week period led to only minor toxicological effects. Moderate inflammation in the lungs was observed at four weeks postexposure without any relation to Zr doping. In contrast, significant inflammation and genotoxicity in the lungs of primarily rats, but also mice, were observed in other studies on the inhalation of CeO_2_ MNMs from various sources [23,24,25,26,27,28,29]. Differences in exposure time, dose and the physico-chemical properties of the MNMs might explain the discrepancies in these results.

TiO_2_ NPs were also used in this study, since a large amount of data is already available from in vivo and in vitro submerged experiments, while there are only a few studies employing ALI exposure [30,31,32]. Inhalation of TiO_2_ MNMs at high concentrations and for long periods triggers inflammation, fibrosis and tumors in the rodent lung [33,34,35]. Recently, Relier et al. studied the genotoxic effects of TiO_2_ MNMs after instillation in rats. While they observed no adverse toxic or genotoxic effects at a low dose (0.5 mg/kg), significant cytotoxicity, inflammation, oxidative stress and DNA damage were observed at overload conditions (10 mg/kg) [36].

Here, non- and redox-modified CeO_2_ NPs were tested under submerged conditions in human A549 lung epithelial cells and differentiated THP macrophages. As read outs, cell membrane damage, metabolic activity and cytokine release were studied. For comparison, A549 cells were also exposed at the ALI depositing low doses matching those investigated in animal experiments. Similarly, A549 cells were exposed to TiO_2_ NPs at the ALI or under submerged conditions. As adverse effects were more pronounced in the case of titania NPs, a more detailed analysis of pro-inflammatory gene expression was performed. Finally, the genotoxicity of TiO_2_ NPs was compared under both exposure methods. The results obtained employing submerged and ALI exposure are discussed critically, specifically considering the dose and relevant in vivo studies. 

## 2. Materials and Methods 

### 2.1. Nanomaterials

The NPs used in this study, as well as their physico-chemical properties, are listed in Table 1. Based on the hypothesis that the redox potential is a driver of the possible toxicity of CeO_2_ NPs, a series of zirconium-modified CeO_2_ NPs with increasing Zr content was prepared. Doping with Zr was achieved by incorporating ZrO_2_ into the cerium oxide crystalline structure, thus altering the redox potential of the cerium NPs. CeO_2_-plain and Zr-doped CeO_2_ MNMs were used as suspensions, and were synthesized using a continuous-flow hydrothermal method which was previously described in the literature [37,38]. The TiO_2_ NPs (AEROXIDE^®^, P25) were kindly provided as a powder by Evonik Industries (Frankfurt, Germany). This material is also named NM-105 in the Nanomaterial Testing Sponsorship Program of the Organization for Economic Cooperation and Development (OECD). Detailed characteristics of the material are published in Rasmussen et al. [39] (see also Table S1). 

### 2.2. Characterization of MNMs in Suspension

To test the particle agglomeration behavior in a cell culture medium by dynamic light scattering (DLS), CeO_2_ and TiO_2_ NPs were diluted in deionized water at 1 mg/mL and ultrasonicated in the Bandelin Sonorex ultrasonic water bath (Bandelin, Berlin, Germany) for 5 min at high frequency power of 120 W_eff_. The stock solutions were further diluted to 125 µg/mL in RPMI1640 cell culture medium supplemented with 100 U/mL penicillin and 100 mg/mL streptomycin, but without serum, to the desired concentrations. The samples were then analyzed by DLS directly (0 h) and after incubation at 37 °C, 5% CO_2_ and 95% rel. hum. for 24 h immediately after vortexing using the Zetasizer Nano ZS (Malvern Instruments Ldt., Herrenberg, Germany) at 25 °C.

The particle suspensions which were used for aerosol generation were also analyzed by DLS using the Horiba LB-500 (Horiba, Sulzbach, Germany). The CeO_2_ NPs were diluted 1:10 in deionized sterile water (CeO_2_-A: 2.37 mg/mL, CeO_2_-C: 2.3 mg/mL, CeO_2_-E: 1.8 mg/mL) and the TiO_2_ NPs were analyzed at 1 mg/mL in water. 

The particles were tested for endotoxin contamination using the chromogenic endpoint Limulus Amebocyte Lysate (LAL) assay (ThermoFisher Scientific, Dreieich, Germany, cat no 88282). Particle suspensions of 1 mg/mL were prepared in deionized water and centrifuged at 20,800× *g* for 10 min. The supernatants were tested for endotoxin content according to the instructions of the manufacturer. The endotoxin content of each NP used was below the lower limit of quantification (0.1 EU/mg).

### 2.3. Determination of the Effective Particle Density and the Relevant In Vitro Dose (RID)

To calculate the RID after submerged exposure, the effective density of the particle agglomerates in the respective media was determined by the volumetric centrifugation method (VCM), as described in DeLoid et al. [40,41]. Briefly, the respective particle suspension was prepared at 125 µg/mL as described under “Hydrodynamic diameter”, and 1 mL was transferred into TPP packed cell volume (PCV) tubes (TPP Techno Plastic Products AG, Trasadingen, Switzerland, cat no 87005) in triplicate and centrifuged in a swinging bucket rotor at 3000× *g* for 1 h. The volume of the particle pellet was determined in µL using a measuring devise from the manufacturer of the PCV tubes. The effective density of the agglomerated particles (Table 1) and the RID delivered to the cells under submerged conditions were then calculated according to the distorted grid (DG) nanotransport simulator as described in DeLoid et al. [41,42,43] on the basis of data on hydrodynamic size, the effective density and other parameters (viscosity of the medium: 0.00074 mPa·s, temperature: 37 °C, CeO_2_ density 7.22 g/cm^3^, TiO_2_ density: 4.23 g/cm^3^, column height: 3.295 mm, concentration of the material: 0.125 mg/mL) in the respective media. Given the high density and size of the CeO_2_ and TiO_2_ NPs, the calculated RID was equivalent to the administered nominal dose as 100% of NPs were deposited.

### 2.4. Generation of NP Aerosols

The 1:10 diluted CeO_2_ NP suspension was stirred continuously while a piston pump (Desaga KP 2000, Wiesloch, Germany) pulled out 20 mL/h into a two-phase nozzle driven with synthetic air (0.8 bar) (type 970, Duesen-Schlick GmbH, Coburg, Germany) into an aerosol reactor according to VDI 3491, sheet 3 (Figure S1). In the double-walled drying reactor, the humidity was removed via diffusional drying using silica gel orange (ThoMar OHG, Luetau, Germany), and then the aerosol was led to the VITROCELL^®^ Automated Exposure Station (VITROCELL Systems, Waldkirch, Germany), which is described in Mülhopt et al. [14]. The TiO_2_ NP powder was weighed, suspended at 1 mg/mL in deionized water and further treated as described above for the CeO_2_ NPs.

Within the reactor of the VITROCELL^®^ Automated Exposure Station, the aerosol was humidified to 85% r.h. and the particle number concentration was measured by a condensation particle counter (CPC) 3775 (TSI Inc., St Paul, MN, USA). The particle size distribution was monitored by a scanning mobility particle sizer (SMPS) 3775 with a differential mobility analyzer (DMA, model 3071) (TSI Inc., St Paul, MN, USA).

### 2.5. Cell Culture and Submerged Exposure

A549 human alveolar epithelial cells (ATCC, Rockville, MD, USA, cat no CCL-185) were used for submerged and ALI exposure. The cells were cultivated in RPMI1640 medium supplemented with 10% FBS (fetal bovine serum), 100 U/mL penicillin and 100 mg/mL streptomycin (Pen/Strep) in 5% CO_2_ at 37 °C (cell culture medium and supplements from ThermoFisher Scientific, Dreieich, Germany). For submerged exposure, 1 × 10^5^ A549 cells were seeded per well of a 24-well plate and treated the next day with different concentrations of particles for 24 h in the absence of FBS [44]. As high levels of serum proteins (e.g., 10% FBS) do not reflect the physiological conditions in the lung, and rather suppress adverse effects due to formation of a protein corona [44], FBS was excluded in the submerged exposure as well as during ALI exposure. Cultivation of THP-1 cells is described in Supplementary data. The CeO_2_ and TiO_2_ NPs were diluted and sonicated as described above for determining the hydrodynamic diameter. 

For ALI exposure, 4 × 10^5^ A549 cells were seeded onto Corning Costar Transwell^®^ insert membranes (polyester, 0.4 µm pore size, surface area 4.67 cm^2^) from Fisher Scientific (Schwerte, Germany, cat no 10619141) and incubated overnight. Before ALI exposure, the apical and the basolateral media were removed. Then, 1.5 mL RPMI 1640 medium without FBS, supplemented with 25 mM HEPES (ThermoFisher Scientific, Dreieich, Germany) and Pen/Strep, was added into the basolateral compartment and the apical side was left uncovered (no medium). 

Here, we used rather low doses of approximately 0.2 and 1 µg/cm^2^ for ALI exposures, as they match with those administered in vivo [36,45]. In submerged conditions, usually much higher doses are deposited; therefore, in order to better compare our findings to the literature, we not only deposited low doses, but also increased the dose up to roughly 40 and 80 µg/cm^2^ for CeO_2_ and TiO_2_ NPs, respectively (for an overview see Table S2).

### 2.6. ALI Cell Exposure

The humidified aerosol was led to the exposure modules where the cells were located and guided through the aerosol inlet tubes towards the cell surface, where the aerosol was brought into direct contact with the cells at the ALI as described previously [14]. The aerosol flow to the cells was 100 mL/min. To enhance deposition of particles, an electrostatic potential between the cells and the aerosol inlet (−1000 Volts) was applied.

The cells were prepared as described above, transported to the ALI exposure system and exposed to “clean air” and to the different NP aerosols at low dose without electrostatic deposition and at high dose with electrostatic deposition for 4 h (see Table 2). For exposure to the more toxic TiO_2_, we also used shorter exposure times, i.e., 30 min, followed by a 3 h 30 min postexposure recovery period. During the postexposure period, the cells remained under ALI conditions in the exposure system receiving particle-free humidified air. Control samples were left in an incubator at 37 °C without CO_2_ supply. After exposure, the medium below the Transwell inserts was collected for LDH analysis. 

### 2.7. Determination of Deposited Dose after ALI Exposure

The mass concentration of the aerosol was determined from the SMPS data and the material densities in Table 1. The dose without electrostatic field (EF) was estimated on the basis of fluorescein deposition. For the dose obtained with electrostatic field (−1000 V), an enhancement factor was applied [13].

The deposited particle dose was additionally calculated from image analysis of particles deposited on grids for transmission electron microscopy (TEM) placed on the Transwell membranes [46]. The copper grids with formvar film (Plano GmbH, Wetzlar, Germany) were exposed in parallel to the cell cultures, but without liquid beneath the membrane. For electrostatic deposition, the Transwell membrane was repositioned to achieve the same electric field strength as in the cell modules. Images were taken using the EM 109T (Carl Zeiss Microscopy GmbH, Oberkochen, Germany) and analyzed for particle number per area using the software ImageJ (version 1.52a, National Institutes of Health, USA, https://imagej.nih.gov/ij/) as described in Mülhopt et al. [14]. The mass per area was calculated by using the same material density as in the SPMS data evaluation.

### 2.8. LDH Release

After particle treatment under submerged conditions or after ALI exposure, the collected medium was centrifuged at 400× *g* to remove cell debris and particles. 100 µL of the supernatant (submerged exposure) or basolateral (ALI exposure) medium was used for quantification of released lactate dehydrogenase (LDH), an indicator of plasma membrane integrity. The LDH assay was performed in accordance with the manufacturer’s instructions (Sigma-Aldrich, Taufkirchen, Germany, cat no 11644793001). As a positive control, nontreated control cells were lysed with 0.1% Triton-X 100 for 30 min prior to the end of the experiments to obtain reference values for the highest LDH release achievable, and the measured values were set to 100%. The absorbance of the reaction mix was measured at 490 nm with a microplate reader and values were analyzed with the software package SoftMaxPro (Molecular Devices, Ismaning, Germany) [44]. No interference with the assay was observed for TiO_2_ and CeO_2_ NPs at the concentrations relevant to our studies [8,30].

### 2.9. AlamarBlue^®^ Reduction

After submerged treatment with CeO_2_ NPs, the supernatant medium was replaced by AlamarBlue^®^ (Bio-Rad Laboratories, Feldkirchen, Germany, cat no BUF012B), diluted 1:10 (*v*/*v*) in RPMI1640 without FBS. The nonfluorescent dye resazurin was converted by mitochondrial dehydrogenases to the fluorescent product resorufin. After 1 h, 100 µL of the supernatant was transferred to 96-well plates and the fluorescence was quantified with a microplate reader (Bio-Tek FL600, software package KC4, MWG-Biotech AG, Ebersberg, Germany) at 580 nm excitation and 620 nm emission. The samples were normalized to the untreated controls (negative control), which were set to 100% [44]. As a positive control, nontreated control cells were lysed with 0.1% Triton-X 100 for 30 min prior to the end of the experiments resulting in a complete loss of the signal (data not shown). No interference with the assay was observed for TiO_2_ (as also reported previously in [8] and CeO_2_ NPs at the concentrations relevant to our studies. To this end, AlamarBlue^®^ reagent was added to cells as described above and after conversion to resorufin no decrease in the fluorescence signal could be detected in the presence of NPs. Furthermore, incubation of the AlamarBlue^®^ reagent with TiO_2_ and CeO_2_ NPs (250 and 125 µg/mL, respectively) in the absence of cells did also not reveal any adsorption of the dye to the NPs.

### 2.10. MTS Reduction 

The viability of the cells treated under submerged conditions with TiO_2_ NPs was also determined using the CellTiter 96^®^ AQueous One Solution Cell Proliferation Assay (MTS, Promega, Walldorf, Germany, cat no G5421) according to manufacturer’s specifications. The reagent contains a tetrazolium compound: 3-(4,5-dimethylthiazol-2-yl)-5-(3-carboxymethoxyphenyl)-2-(4-sulfophenyl)-2H-tetrazolium, inner salt MTS. Briefly, MTS is reduced by metabolically active cells into a colored formazan product that is soluble in tissue culture medium. The quantity of the formazan product, as measured by the absorbance at 490 nm, is directly proportional to the bulk metabolic activity of cells in culture. After exposure, the culture medium was removed and the MTS reagent was applied for 1 h and the supernatant was transferred to a 96-well plate to measure the absorbance at 490 nm [47]. The samples were normalized to the untreated controls (negative control), which were set to 100%. As a positive control, nontreated control cells were lysed with 0.1% Triton-X 100 for 30 min prior to the end of the experiments resulting in a complete loss of the signal (data not shown). No interference with the assay has been observed for TiO_2_ (in accordance with [48]) and CeO_2_ NPs at the concentrations relevant to our studies. To this end, MTS reagent was added to cells as described above and after conversion to the formazan product no decrease in the signal could be detected in the presence of NPs. Furthermore, incubation of the MTS reagent with TiO_2_ and CeO_2_ NPs (250 and 125 µg/mL, respectively) in the absence of cells did also not reveal any adsorption of the dye to the NPs.

### 2.11. IL-8 Release

The secreted IL-8 was analyzed in the cell culture medium (for submerged exposures) using the OptEIA ELISA kit (Becton Dickinson, Heidelberg, Germany, cat no 555244) according to the manufacturer’s instructions. For measurement of absorption and data analysis, a microplate reader and the software package SoftMaxPro (Molecular Devices, Ismaning, Germany) were used [44]. As a positive control, lipopolysaccharide (LPS from *E. coli*, Sigma-Aldrich, Taufkirchen, Germany, cat no L2630) was added (10 µg/mL). No interference with the assay has been observed for TiO_2_ and CeO_2_ NPs at the concentrations relevant to our studies [30].

### 2.12. RT-qPCR 

The expression of genes encoding enzymes implicated in cell redox balance reestablishment, as well as cytokines, was measured by RT-qPCR (reverse transcription-quantitative polymerase chain reaction). Cells were exposed to NPs, and then washed three times with PBS. mRNAs were extracted and reverse-transcribed. qPCR was ran using a Stratagene MX3005P (Agilent, Santa Clara, CA, USA) using the following thermal cycling steps: 95 °C for 5 min, then 95 °C for 15 s, 55 °C for 20 s and 72 °C for 40 s 40 times and finally 95 °C for 1 min, 55 °C for 30 s and 95 °C for 30 s to obtain the dissociation curve. Gene expression was normalized to three reference genes: Glyceraldehyde-3-phosphate dehydrogenase (GAPDH), 18S ribosomal 1 (S18) and cyclophilin A (cycloA). The primers used for the RT-qPCR experiments are shown in Table S3. All three reference genes showed standard deviations of less than 1 and a strong correlation with the BestKeeper Index. Gene expression analysis, normalization and statistical analysis were performed with REST 2009 software using the ΔΔCq method and a pair-wise fixed reallocation randomization test.

### 2.13. Detection of DNA Strand Breaks and Alkali-Labile Sites

DNA strand breaks and alkali-labile sites were assessed through the alkaline version of the comet assay and Fpg-sensitive sites, including 8-oxo-dGuo, were quantified by using the bacterial DNA repair enzyme formamidopyrimidine-DNA glycosylase (Fpg) as described previously [49]. Briefly, at the end of the ALI exposure, the cells were detached with trypsin, centrifuged at 250− *g* for 5 min, suspended in the storage buffer, composed of sucrose 85.5 g/L, DMSO 50 mL/L prepared in citrate buffer (11.8 g/L), pH 7.6, and immediately frozen at −80 °C. For the comet assay six microscope slides per condition were coated with 1% normal melting point agarose (NMA) and allowed to dry. 10,000 cells per slide were mixed with 0.6% low melting point agarose (LMPA) and deposited over the NMA layer. The cell/LMPA mix was then allowed to solidify on ice for 10 min. Slides were immersed in cold lysis solution (2.5 M NaCl, 100 mM EDTA (ethylenediamine tetraacetic acid), 10 mM Tris, 10% DMSO (dimethyl sulfoxide), 1% Triton X-100) overnight at 4 °C, before being rinsed in PBS. Then 3 slides were treated with 100 μL Fpg (5 U/slide, in enzyme buffer, Trevigen, Gaithersburg, MD, USA) and 3 slides were incubated with Fpg enzyme buffer for 45 min at 37 °C. DNA was then allowed to unwind for 30 min in alkaline electrophoresis solution (300 mM NaOH, 1 mM EDTA, pH > 13). All chemicals were from Sigma-Aldrich (Saint-Quentin-Fallavier, France).

Electrophoresis was performed in an electric field of 0.7 V/cm and 300 mA for 30 min. Slides were then neutralized in PBS and were stained with 50 μL of 20 mg/mL ethidium bromide (Life Technologies, Carlsbad, CA, USA). At least 50 comets per slide were analyzed under a fluorescence microscope (Carl Zeiss, Oberkochen, Germany) connected to a charge-coupled device camera with a 350–390 nm excitation and 456 nm emission filter, at 20× *g* magnification. Comets were measured and analyzed using Comet IV software (Perceptive Instruments, Suffolk, UK). As a positive control 50 µM H_2_O_2_ was used.

### 2.14. Statistics

Results are reported as mean ± standard error of the mean (s.e.m.) or standard deviation (SD) of multiple independent experiments. Statistical significance was tested using Student’s t-test or Kruskall-Wallis nonparametric one-way analyses of variance by ranks, using Statistica 8.0 software (Statsoft). When significance was demonstrated (*p* < 0.05), paired comparisons were run using Mann-Whitney *u*-tests.

## 3. Results

### 3.1. Particle Characterization

The physico-chemical properties of the unmodified CeO_2_-A (0% Zr), the redox-modified CeO_2_-C (27% Zr) and CeO_2_-E (78% Zr) and the TiO_2_ NPs are given in Table 1. The CeO_2_ and TiO_2_ NPs agglomerated slightly in water, showing z-average values between 44.3 to 165 nm. However, all NPs strongly agglomerated when suspended in cell culture medium without FBS.

### 3.2. Aerosol Characterization

**Figure 1:**
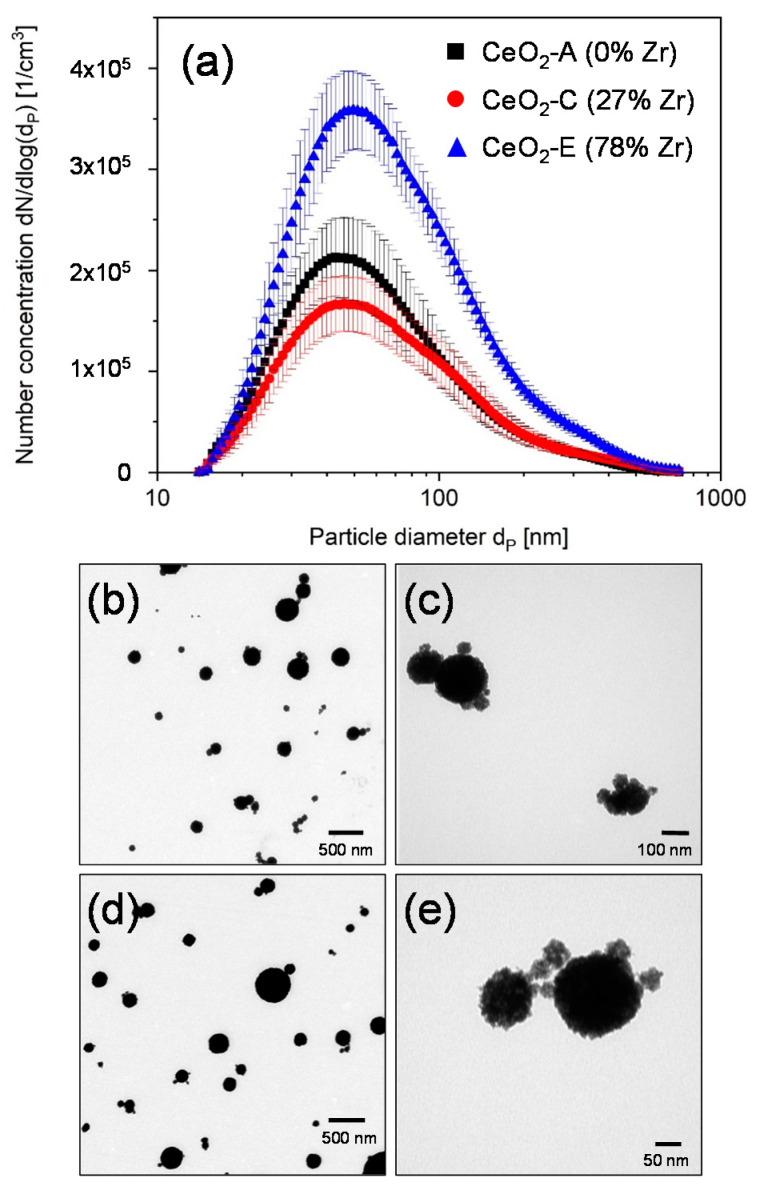
Aerosol characteristics and deposition of CeO_2_ NPs. (**a**) Number-size distribution of the three different CeO_2_ modifications measured in the conditioning reactor of the exposure system. (**b**–**e**) In experiments without cells, the particles were deposited on ALI exposed grids located on the Transwell inserts for 4 h without and for 2 h with electrostatic field (EF). (**b**,**c**) TEM images with two different magnifications of CeO_2_-A NPs deposited by diffusion. (**d**,**e**) TEM images of CeO_2_-A NPs deposited with EF.

**Figure 2:**
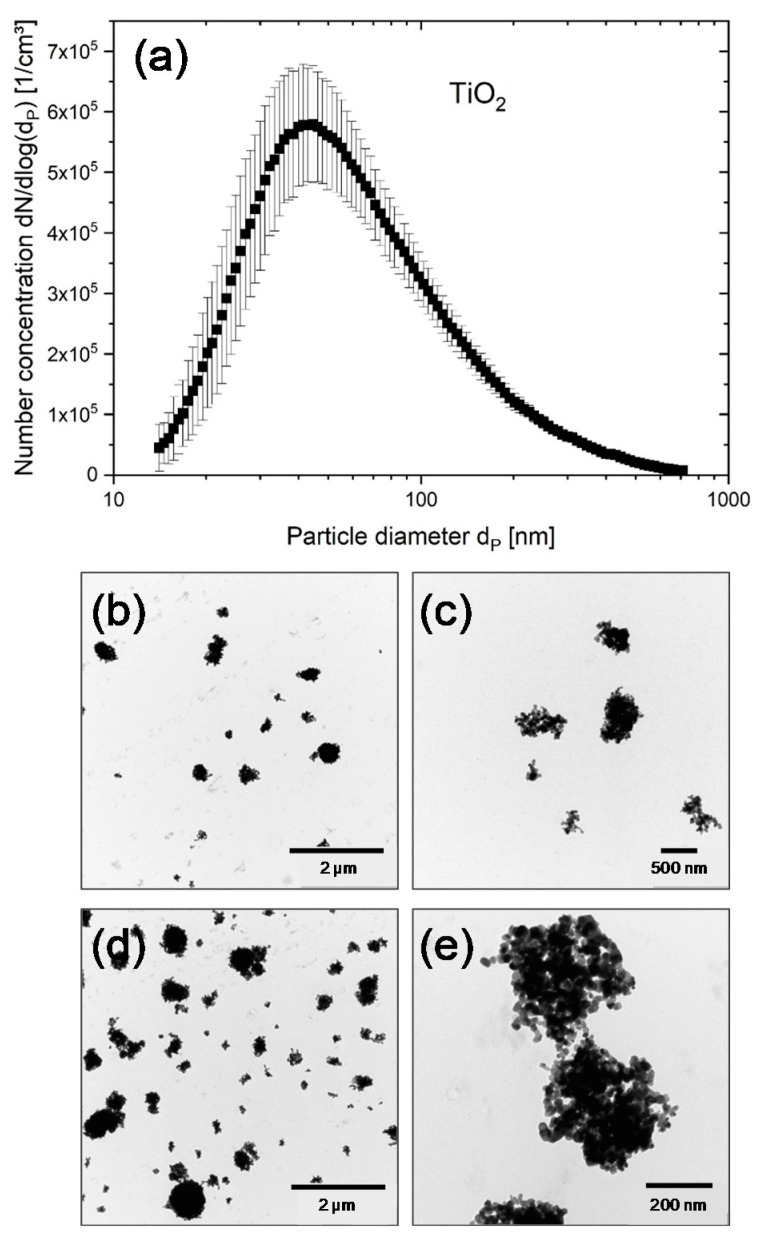
Aerosol characteristics and respective deposition of TiO_2_ NPs. (**a**) Number-size distribution measured in the conditioning reactor of the exposure system. (**b**–**e**) In experiments without cells, the particles were deposited on ALI exposed grids located on the Transwell inserts for 4 h without and for 2 h with electrostatic field (EF). (**b**,**c**) TEM images of TiO_2_ NPs with two different magnifications deposited by diffusion. (**d**,**e**) TEM images of TiO_2_ NPs with two different magnifications deposited with EF.

The particle mass concentration in the aerosol was calculated according to the material density of the particles and the particle number distribution measured by SMPS (Table 2). Figure 1a and Figure 2a show the number size distributions of the humidified CeO_2_ and TiO_2_ aerosols, respectively, measured by SMPS inside the conditioning reactor. There was a slight variation in total number concentration of the CeO_2_ aerosols from 1.3 × 10^5^/cm^3^ (CeO_2_-C) to 2.2 × 10^5^/cm^3^ (CeO_2_-A), while the modal diameter remains about the same, namely 49 nm (CeO_2_-A), 52 nm (CeO_2_-C) and 48 nm (CeO_2_-E) (Table 2). Compared to the nominal primary particle diameter of 20 nm, the aerosolized particles seemed to agglomerate slightly. Therefore, TEM images were taken in a separate experiment under identical conditions as used for the ALI experiments with cells to corroborate the size of the deposited particles as well as the relative mass increase in case of electrostatic compared to diffusional deposition (Figure 1b–e). Calculations were performed as described in Mülhopt et al. [14]. Images of deposited TiO_2_ NPs were taken at both conditions. Results are shown in Figure 2b–e for diffusional and for electrostatic deposition, respectively. The number-size-distribution of the TiO_2_ aerosol (Figure 2a) showed that the modal particle diameter was in the nanometer range (47 nm ± 3 nm). The doses for different time points of exposure were estimated as for the CeO_2_ MNMs (Table 2). It is demonstrated that there is a linear increase of dose with exposure time (as determined for titania) and a 5–7-fold enhanced deposition of NPs by application of the electrostatic field. 

### 3.3. Submerged Exposure to MNMs 

**Figure 3:**
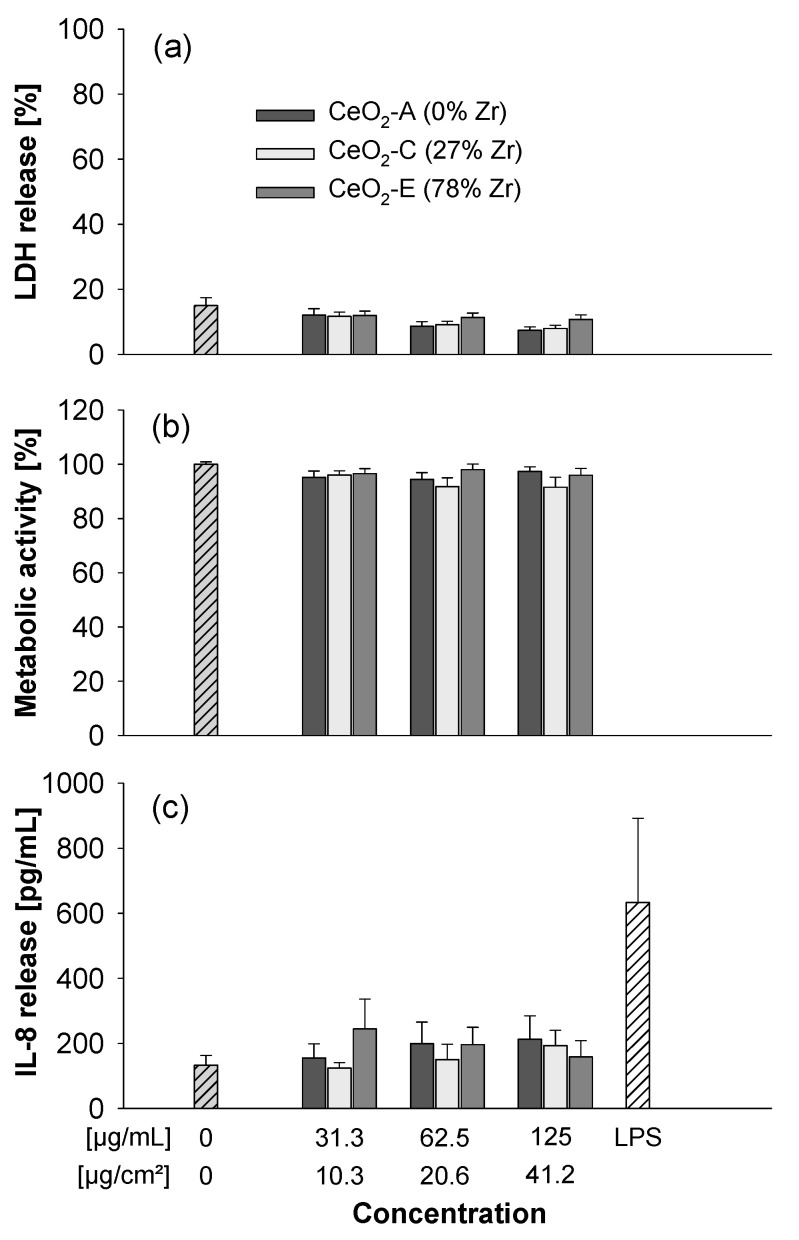
Submerged exposure to non- or redox-modified CeO_2_ NPs triggers no adverse effects. A549 cells were either left untreated or exposed at the indicated concentrations of CeO_2_ NPs suspended in medium without FBS for 24 h. 10 µg/mL LPS served as positive control for IL-8 release. The LDH release was analyzed in the medium (**a**) and is shown as percentage of the positive control (Triton-lysed cells set to 100%). The AlamarBlue reduction (**b**) reflecting the metabolic activity of the cells was normalized to untreated control cells. Data on IL-8 release is shown in (**c**). The data represent mean values of three independent experiments performed in duplicate ± s.e.m.

**Figure 4:**
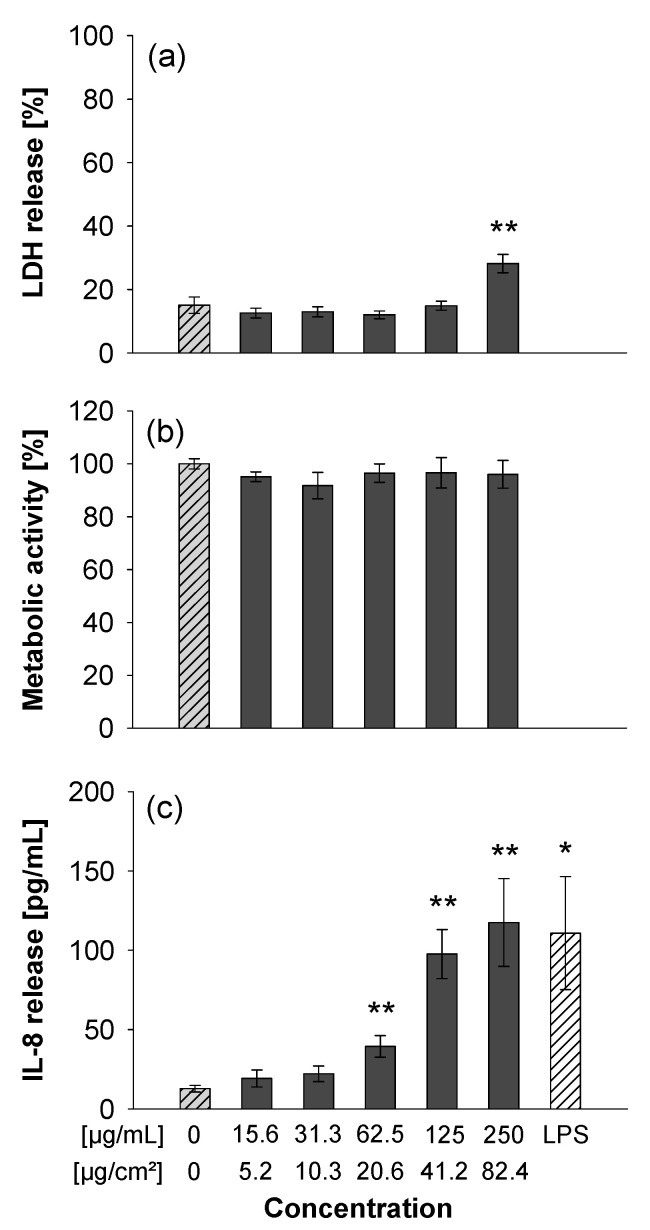
Submerged exposure to TiO_2_ NPs provokes IL-8 release. A549 cells were exposed as described in Figure 3. The LDH release was analyzed in the medium (**a**) and is shown as a percentage of the positive control (Triton-lysed cells, 100%). The MTS reduction (**b**) which monitors the metabolic activity of the cells was normalized to untreated control cells. Data on IL-8 release is shown in (**c**). The data represent mean values of three independent experiments performed in duplicates ± s.e.m. * *p* < 0.05 and ** *p* < 0.01 indicate significant differences in the response of treated cells compared to those incubated only with medium (0).

The CeO_2_ and Zr-doped CeO_2_ NPs were tested in A549 cells for cytotoxicity and cytokine release at concentrations up to 125 µg/mL. None of the particles induced membrane damage (as assessed by release of LDH) or impacted metabolic activity (measured by the AlamarBlue assay). The particles also did not provoke a release of the cytokine IL-8 which is an indicator of a pro-inflammatory response (Figure 3). In addition, differentiated THP-1 macrophages were exposed to the different ceria NPs. Yet again as observed for the experiments with the A549 cells, no clear adverse effects were detected (Figure S2). Similarly, the TiO_2_ NPs were not cytotoxic up to 125 µg/mL, yet they triggered some LDH release at 250 µg/mL (Figure 4). However, the titania particles clearly induced a dose-dependent release of IL-8 (Figure 4c).

### 3.4. ALI Exposure to NP Aerosols

**Figure 5:**
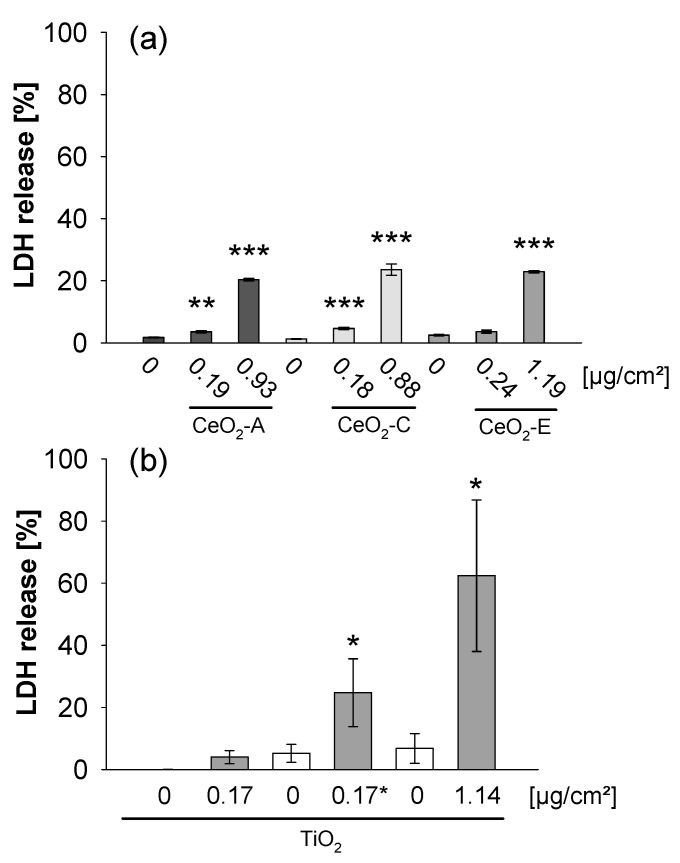
ALI exposure to CeO_2_ (**a**) and TiO_2_ NPs (**b**) triggers cytotoxicity in A549 cells. The characteristics of the aerosols generated from the NP suspension and the estimated doses are shown in Table 2. The cells were exposed to CeO_2_ NP aerosol for 4 h without EF and with EF (−1000 V) to deposit a low and a high dose as indicated. Similar doses of TiO_2_ NPs were deposited in presence of an EF (−1000 V) for 30 min + 3 h 30 min recovery simply at the ALI without further exposure to TiO_2_ NPs to achieve a low exposure dose (0.17 µg/cm^2^; * recovery period indicated by an asterisk) and for 4 h of constant aerosol exposure to yield a high exposure dose (1.14 µg/cm^2^), respectively. To evaluate the effect of exposure time on toxicity, cells were exposed to TiO_2_ NPs only for 30 min and directly analyzed (0.17 µg/cm^2^). For comparison, controls were exposed to clean humidified air (Air). The LDH release was analyzed in the medium after ALI exposure and is shown as percentage of the positive control (Triton-lysed cells set to 100%). The data are means of three (CeO_2_-A, CeO_2_-C, TiO_2_) and two (CeO_2_-E) independent experiments, respectively, performed in triplicates ± s.e.m. * *p* < 0.05, ** *p* < 0.005, and *** *p* < 0.001 indicate significant differences in the response of treated cells compared to controls.

After exposure to CeO_2_ NPs at the low dose (~0.2 µg/cm^2^) (Figure 5a), there was a slight increase of LDH released from A549 cells compared to the clean air controls. However, at the high dose (~1 µg/cm^2^), CeO_2_ NPs, independent of Zr-doping, clearly triggered toxicity, as evidenced by roughly 12-fold (CeO_2_-A), 19-fold (CeO_2_-C) and 9-fold (CeO_2_-E) inductions of LDH release. In contrast, submerged exposure of A549 cells to much higher doses (10.3, 20.6 and 41.2 µg/cm^2^) of CeO_2_ NPs did not enhance LDH release (Figure 3), indicating that cells exposed at the ALI were much more sensitive compared to submerged cultures. 

The cytotoxic effect of TiO_2_ NP aerosol in A549 cells was already detected at ~0.2 µg/cm^2^, when particles were deposited for 30 min followed by 3.5 h postincubation at the ALI (Figure 5b). Of note, 30 min exposure at the same dose without postincubation did not impair membrane integrity, suggesting a delayed detrimental impact of particles. Increasing the dose to ~1 µg/cm^2^ enhanced membrane damage even more drastically. Again, as observed in case of ceria, the adverse effects observed at the ALI were not detected under submerged conditions up to a concentration of ~40 µg/cm^2^, and started to become significant only at the highest dose, i.e., ~80 µg/cm^2^ (Figure 4).

**Figure 6:**
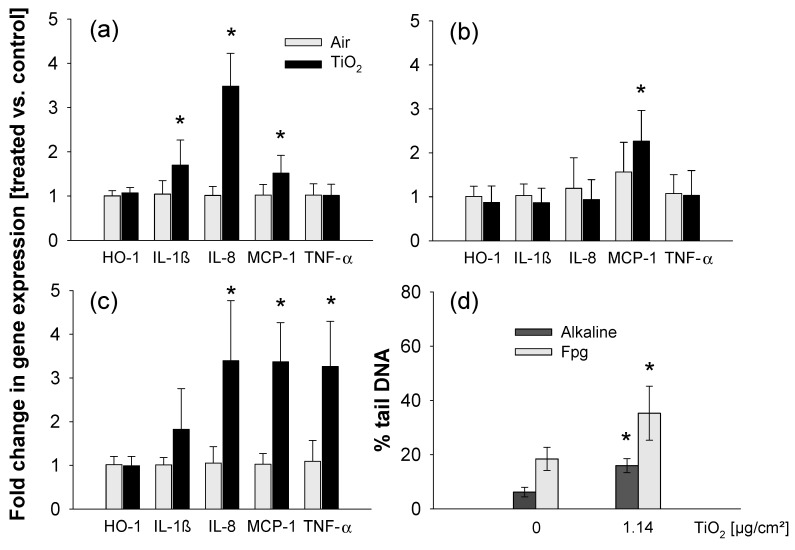
Induction of pro-inflammatory genes and genotoxicity in A549 cells exposed to TiO_2_ NPs at the ALI. Gene expression was measured by RT-qPCR on samples from three independent exposure experiments. Exposure times were 30 min (**a**), 30 min followed by a 3 h 30 min recovery period without exposure to TiO_2_ NPs (**b**) or 4 h (**c**) to deposit 0.17 µg/cm^2^ (**a**,**b**) and 1.14 µg/cm^2^ (**c**), respectively. Data are mean values of fold change of treated vs. control (clean air) samples ± SD of three independent experiments. Statistics * *p* < 0.05, exposed vs. control. (**d**) Cells were exposed to clean air or to TiO_2_ NPs for 4 h at 1.14 µg/cm^2^. The TiO_2_ exposed cells were analyzed directly after 4 h ALI exposure. DNA strand breaks were analyzed by the comet assay in its alkaline and Fpg-modified versions. Depicted are means of three independent experiments ± SD. * *p* < 0.05, treated vs. controls.

As under submerged conditions, titania NPs provoked release of IL-8 (interleukin-8, chemoattractant which attracts neutrophils) (Figure 4), the mRNA levels of IL-8 and additional pro-inflammatory and stress markers i.e., TNF-α (tumor necrosis factor-alpha, early marker of pro-inflammatory response), IL-1β (interleukin-1 beta, marker of pro-inflammatory response), HO-1 (heme oxygenase-1, marker of oxidative stress) and MCP-1 (monocyte chemotactic protein-1, chemoattractant which attracts macrophages) were analyzed (Figure 6). Cells were analyzed just after 30 min or 4 h exposure. Furthermore, cells exposed for 30 min to titania NPs and postincubated at the ALI for another 3 h 30 min were also studied to address the impact of kinetics on the response.

Exposure to an aerosol of TiO_2_ NPs for 30 min and a dose of ~0.2 µg/cm^2^ induced the expression of the pro-inflammatory cytokine IL-1β and the chemoattractant MCP-1 rather moderately whereas IL-8 mRNA levels were clearly elevated (Figure 6a). Interestingly, additional postincubation at the ALI for 3.5 h blunted the effects (Figure 6b). Furthermore, 4 h exposure at a dose of ~1 µg/cm^2^ not only led to induction of IL-1β, MCP-1 and IL-8, but also of TNF-α (Figure 6c). In contrast, enhanced expression of the oxidative stress marker HO-1 upon exposure to TiO_2_ was not observed under any of the monitored conditions, despite the pronounced and dose dependent formation of ROS by the TiO_2_-NPs as validated in vitro (Figure S3). Remarkably, in cells exposed for 4 h to similar concentrations of TiO_2_ NPs under submerged conditions, mRNA levels of IL-8 were not significantly increased (Figure S4). 

Damage to DNA caused by theseTiO_2_ NPs was then assessed using the comet assay in its alkaline and Fpg-modified versions, probing the presence of single and double strand breaks and alkali-labile sites (alkaline) and of Fpg-sensitive sites such as 8-oxo-dGuo, a marker of oxidative DNA damage (Fpg-modified version). A significant increase of strand breaks and alkali-labile sites was observed in cells exposed to TiO_2_ NPs at the ALI at a dose of ~1 µg/cm^2^ (Figure 6d). Immunostaining and counting of 53BP1 foci showed a significant increase in the number of foci after ALI exposure (Figure S5a), proving that at least part of this DNA damage was due to double-strand breaks (DSBs). This increase was significant only after 4 h but not after 30 min exposure, suggesting an indirect mechanism of genotoxicity, which could be via impairment of DNA repair activities, as already suggested for studies with these NPs after exposure under submerged conditions at high concentration [50,51].

Under submerged conditions, no DNA damage was observed at similar concentrations as in the ALI exposure, but started to occur only at a 10-fold higher concentration (Figure S5b). Likewise, under submerged exposure conditions, no significant increase of 53BP1 foci was observed (Figure S5c). Again, similar to the cytotoxic and pro-inflammatory response the genotoxic effects of titania NPs are more pronounced upon ALI exposure compared to exposure under submerged conditions. 

Finally, we compared the responses observed in our in vitro studies with recent in vivo experiments investigating the same titania NPs upon tracheal instillation in rats [36]. For the endpoints cytotoxicity, genotoxicity and inflammation lowest observed adverse effect levels (LOAELs) could be determined (Table 3). Intriguingly, the LOAELs derived from the in vivo studies are in a similar dose range as those established for the ALI experiments, whereas in the case of submerged exposures LOAELs are much higher or could not be defined.

## 4. Discussion

The development of advanced ALI exposure systems contributes to the establishment of predictive in vitro tests which would simulate the in vivo situation during inhalation much more accurately than available submerged assays [3,6,14]. Currently, in vivo experiments are still the standard for regulatory testing under REACH following established OECD guidelines. Recently, 19 chemicals with known toxicities specifically to the lung were studied regarding their CLP (Classification, Labelling and Packaging of substances and mixtures) classification for acute inhalation toxicity by assessment of cell viability after ALI exposure in a CULTEX system employing A549 cells [52]. A comparison to submerged exposure experiments revealed a higher sensitivity of cells exposed at the ALI. Apart from simple monocultures, more complex cocultures including primary bronchial cells derived from healthy or diseased donors are also used to monitor acute inflammatory responses at the ALI and to address the enhanced sensitivity of vulnerable cohorts such as asthmatic patients towards particle exposure [53]. Combinations of more sophisticated biological systems with advanced and more comprehensive monitoring of adverse effects by OMICS analysis make it possible to investigate not only individual chemicals or particles at the ALI, but also complex mixtures such as combustion derived aerosols [15,16,53,54,55].

For a number of MNMs, mostly metal and metal oxide NPs, but also carbon nanotubes, in vitro experiments were performed and the outcomes were contrasted with in vivo data considering the applied dose [3,45]. However, comparisons are often difficult due to the different nominal doses which are usually much higher in submerged experiments. Moreover, the delivered cellular dose in such submerged in vitro studies often remains elusive. Although at the ALI the deposited dose is much better defined, the dose rate often varies drastically between the different exposure systems. Bolus (within seconds to minutes) versus linear (several hours) application of particles might trigger totally different biological responses due to exhaustion of cellular defense mechanisms and therefore the outcome of the limited number of ALI studies employing different systems on the toxicity of certain NPs are also not directly comparable. Specifically, for ceria and titania NPs which are abundant MNMs and have been widely studied in vitro and in vivo including ALI experiments [32,56,57,58,59] these experimental differences could impact the final response. Also, multiple procedures for the generation of ceria and titania aerosols, different exposure concentrations, cell culture conditions and types of read-outs were used in published studies, further complicating the interpretation of the various findings. Finally, several types of ceria and titania NPs with distinct physico-chemical properties were investigated. In the following, we will discuss our data on cytotoxicity, pro-inflammatory response and the rarely investigated genotoxicity in A549 and THP-1 cultures triggered by ceria and titania NPs upon exposure at the ALI or under submerged conditions in light of all these parameters. In addition, we analyze the existing in vivo data on pulmonary effects of ceria and titania NPs with particular emphasis on the dose, exposure time and type of read-out to better correlate in vitro with in vivo findings and provide recommendations for future studies. 

### 4.1. Effects of Ceria NPs Studied in In Vitro Experiments under Submerged or ALI Conditions and Comparison to In Vivo Findings

CeO_2_ NPs were selected as acute and subacute toxicity data from inhalation exposure are available [23,24,25,26,27,28,29]. As reviewed in Landsiedel et al. [45], ceria NPs trigger moderate cytotoxicity. This effect was also observed in most studies under submerged conditions; however, cytoprotective effects have also been reported. These discrepancies might be explained by different physico-chemical properties of the various ceria NPs related to surface chemistry and redox activities. 

In our studies, A549 cells did not respond to the different CeO_2_ NPs under submerged conditions, in accordance with other reports in which, for example, LDH was also used as a read-out to monitor toxicity of CeO_2_ NM-212 [24]. While we did not detect any loss of viability (recorded by the LDH and AlamarBlue assays) up to 125 µg/mL, which corresponds to 41.2 µg/cm^2^, Dekkers et al. [22] reported a decrease of A549 viability down to 50–60% upon exposure for 24 h to 40–80 µg/cm^2^ of the same 3 different CeO_2_ NPs, which we also used in our experiments. These contrasting results might be due to the different exposure media (FBS was added in the latter studies whereas we omitted FBS for better comparison with our ALI experiments), the different procedures to measure metabolic activity (WST-1 versus AlamarBlue assay) or to ageing of the NPs during storage. Sauer et al. [60] found that exposure of rat precision-cut-lung slices to CeO_2_ NM-211 or 212 MNMs induced cytotoxicity at 1000 µg/mL, as determined by the WST-1 assay and moderate cytokine release (TNF-α, CINC1 (which corresponds to human IL-8), M-CSF, OPN (Osteopontin)) at nontoxic doses of 100 µg/mL, suggesting that more complex biological systems could be better suited to detect adverse effects of NPs. However, much higher doses are needed to promote toxic responses as compared to in vivo studies [60].

Meanwhile, there are a few published in vitro ALI studies which tested CeO_2_ NPs [30,61,62]. Steiner et al. [63] exposed a 3D coculture model prepared from A549 cells, human monocyte-derived macrophages and dendritic cells under ALI conditions to a CeO_2_ NP suspension (0.75 µg/cm^2^) employing a microsprayer and found increased expression of HO-1, but not of SOD1, TNF-α, IL-8 and no release of LDH. Using a glovebox for exposure of A549 cells, freshly generated ceria NPs were transferred over 30 min at a maximal dose of 24 µg/cm^2^ [57]. Whereas after 24 h, no LDH release could be documented, despite efficient particle uptake [56], reduced volume of lamellar bodies and transepithelial electrical resistance (TEER) accompanied by loss of the tight junction marker occludin was observed [57]. In addition, enhanced levels of 8-oxoguanine, an indicator of oxidative DNA damage, were monitored. However, in another study, A549 cells were exposed to different doses of CeO_2_ up to 100 µg/cm^2^ deposited within 60 min, which induced a significant reduction of cell viability as assessed by WST-1 reduction after 24 h postexposure [32]. Hence, the dose rate (i.e., mass delivered over time) often varies between studies and might be a critical determinant of the ensuing cellular response.

Loret et al. [30] studied the effects of CeO_2_ NM-212 in A549 monocultures and cocultures with THP-1 macrophages after 3 h ALI exposure using a VITROCELL exposure system and 21 h postexposure and compared the results to submerged exposures. Using the ISDD model they calculated the max. dose of 9.5 µg/cm^2^ after 24 h submerged deposition and at the ALI as max. 3.3 µg/cm^2^. CeO_2_ NM-212 had nearly no effects irrespective of the cell culture model or exposure system. These findings are in line with a later report employing an XposeALI system depositing CeO_2_ NM-212 (max. 5 µg/cm^2^) within 5–20 min onto a coculture of A549 and THP-1 where no major effects on cytotoxicity and cytokine release became evident [61]. Similarly, and also using a VITROCELL exposure system, adverse responses were investigated in A549, BEAS-2B (max. 0.71 µg/cm^2^) and MucilAir cells (max. 3 µg/cm^2^) after ALI exposure to CeO_2_ NPs and 24 h postexposure [64]. In MucilAir cells, only an increase of HO-1 protein, but no significant responses with regard to cytotoxic, inflammatory and genotoxic parameters, were found. Conversely, HO-1 was not changed in A549 and BEAS-2B cells. Only BEAS-2B cells moderately responded, with increases in LDH and IL-8 release but in both cell lines genotoxicity was induced as demonstrated by the alkaline comet assay. This data shows that the cell type significantly affects the outcome in ALI exposure studies. Specifically, the MucilAir model seems to be less sensitive compared to cell lines possibly due to enhanced clearance of particles by a mucous layer and ciliary movement.

In our ALI experiments, a CeO_2_ NP dose of approximately 0.2 and 1 µg/cm^2^ over a time frame of 4 h was deposited onto A549 cells. A dose dependent increase in cytotoxicity could be demonstrated which was, however, independent of Zr-doping. Zr was incorporated successfully and the Ce^3+^/Ce^4+^ ratio increased according to the different amounts of Zr as published previously [22]. Nevertheless, enhanced antioxidant properties of the Zr-doped versus unmodified ceria NPs could not be convincingly demonstrated, which likely explains the similar toxicity of all 3 NPs used in our work.

Interestingly, the results of the present study on LDH release correlate well with in vivo results obtained in rats after a short-term inhalation study (STIS) with CeO_2_ NM-212 [27]. Rats were exposed to 0.5, 5 and 25 mg CeO_2_ NPs/m^3^ for 6 h/day for 5 days. The results at 3 days after the end of exposure indicate cytotoxicity (LDH release), pro-inflammatory (neutrophil influx in BALF, CINC1/IL-8, MCP-1) and pro-fibrotic responses (M-CSF and osteopontin release) at the threshold concentration of 5 mg/m^3^. The total lung burden at this concentration was 100 µg (about 0.02 µg/cm^2^). Toxic effects were even more pronounced at 25 mg/m^3^ and a lung burden of about 500 µg resulting in a calculated surface area dose of about 0.1 µg/cm^2^ [45]. Considering the proximal alveolar region (PAR) where particle retention is the greatest, the critical targeted surface area would be roughly 10-fold reduced in the lung and the deposited dose accordingly increased, which yields better correspondence among in vitro and in vivo data [65]. Therefore, at doses which are more similar to the delivered dose in vivo, and which are much lower than those applied in conventional submerged culture experiments, cytotoxicity can be monitored using the ALI system. 

Mechanistically, increased membrane damage observed in ALI experiments and in the lung correlate well with the inflammogenicity observed in vivo and might be the apical event to drive inflammation.

### 4.2. Effects of Titania NPs Studied in In Vitro Experiments under Submerged or ALI Conditions and Comparison to In Vivo Findings

Also, for titania NPs, a number of in vitro and in vivo studies have been performed previously. Cytotoxicity is dependent on the crystal structure (anatase being more potent than rutile), coating as well as size and is largely driven by the generation of ROS due to photoactivation of titania (reviewed in [66]). Toxicity pathways are related to membrane damage and cell death, but also to inflammation, and are critically determined by the investigated cell type [67]. In particular, in A549 cells and RAW264.7 macrophages cultured in submerged conditions titania NPs showed only minor effects [8,67]. Notably, noncytotoxic doses of TiO_2_ NPs induce DNA damage upon long-term exposure [49]. Here, the toxicity of titania P25 whose crystal structure is a mix of anatase and rutile (80/20) was studied. As also published by others [68,69], under submerged conditions at doses exceeding 10 cm^2^ specific NP surface area per cm^2^ cell layer area an increase in IL-8 release in A549 cells was observed but little cytotoxicity was evident. In contrast to many studies on titania NPs performed with classical submerged cultures, only a limited number of ALI experiments have been reported. As already discussed above for ceria NPs, Steinritz et al. exposed A549 cells to max. 100 µg/cm^2^ of titania P25 NPs within 60 min and report a significant reduction of the metabolic activity after 24 h as measured by the WST-1 assay [32]. This was recapitulated in a further study [70], in which an enhanced toxicity of titania NPs versus ceria NPs was noted, which is in accordance with our results obtained after ALI exposure. In contrast, deposition of up to 26 µg/cm^2^ titania NPs onto A549 cells within 10 min in a CLOUD system via microdroplets did not reduce cell counts or impair DNA integrity as assessed by alkaline unwinding after 24 h, nor did it affect the ability to form colonies over 10 days after exposure [58]. As different dose rates, exposure systems and methods for the detection of cytotoxicity were used the reason for the diverging results remain unknown and need to be further investigated. In a very systematic and detailed study A549 cells alone and in coculture with THP-1 macrophages were exposed to TiO_2_ NM-105, NM-101, NM-100 and CeO_2_ NM-212 aerosol at 0.1, 1 and 3 µg/cm^2^ for 3 h and 21 h postincubation [30]. Release of the pro-inflammatory mediators IL-1β, IL-6, IL-8 and TNF-α was only observed in the coculture at the two highest doses of the TiO_2_ NPs while CeO_2_ NM-212 NPs induced only minor effects. Furthermore, effects on cellular integrity i.e., LDH release by titania NPs were negligible. Interestingly, cocultures were more vulnerable to titania NPs than monocultures and effects were observed at lower doses at the ALI than under submerged conditions. Although we also used a similar exposure system, the time point when adverse effects were monitored differed (4 h versus 24 h) which might have an impact on the response detected. Indeed, we could clearly observe pro-inflammatory gene expression in A549 monocultures as well as membrane damage which was dose dependent. Thus, consideration of the kinetics of the various cellular responses would be warranted for future ALI investigations and could further improve the sensitivity of the test system. In agreement with Loret et al. we also confirm an increased response at the ALI at lower doses compared to conventional submerged exposures. In addition, DNA damage is induced at a very low dose of ca. 1 µg/cm^2^ at the ALI but 10-fold higher concentrations are needed under submerged conditions. 

Also, in rodents (primarily rats), numerous adverse effects due to inhalation or instillation of TiO_2_ NPs have been documented [66]. Ma-Hock et al. [34] observed transient inflammation in rat lungs after short-term inhalation (6 h/day for 5 days) of TiO_2_ NPs already at 10 and more pronounced at 50 mg/m^3^ (mean primary size 25 nm, corresponding to up to 0.4 μg TiO_2_/cm^2^ lung tissue [66] but no systemic effects were observed. At the highest dose, enhanced levels of LDH were detected. A study on the effects of TiO_2_ NPs after three instillations over 8 days in rats under overload and nonoverload conditions was performed more recently [36]. The authors observed reduced lung clearance and cell damage at the two higher doses (i.e., 2.5 and 10 mg/kg) exceeding the limits of 200–300 cm^2^ specific NP surface area per lung or 1 cm^2^ specific NP surface area per cm^2^ lung area as also reported by others [65]. Interestingly, when we compared the lowest adverse effect levels (LOAELs) derived for the endpoints cytotoxicity, genotoxicity and inflammation from the in vivo studies with our in vitro findings, the effective dose range was quite comparable in the case of the ALI exposures, but was much higher for the submerged experiments (Table 3). This corroborates and extends a previous publication where A549 cells were cocultured with THP-1 macrophages at the ALI and inflammatory responses could be compared to a short-term (24 h) instillation study in rats [70]. Here, we could further advance this concept and demonstrate that cytotoxicity and genotoxicity can also be predicted by exposing A549 monocultures to titania NPs at the ALI at comparable concentrations to those used in in vivo studies.

Further improvements of ALI exposure systems could entail the use of more complex coculture systems, primary lung cells and longer or repeated exposure times over days or potentially weeks to pave the way to better compare in vitro findings at the ALI with prolonged exposure studies in vivo and to assess the predictivity of the ALI exposure method [71,72,73,74]. Indeed, Chortarea et al. [75,76] have already demonstrated that repeated ALI exposure is possible over 5 weeks/5 days per week using normal and asthmatic primary human bronchial epithelial cells (MucilAir) which were exposed to multiwalled carbon nanotubes and DQ12 quartz to assess adverse effects. Thus, ALI exposure systems should be more broadly employed and further developed to investigate pulmonary toxicants, as this technology holds great promise to refine, reduce or even replace animal experiments, hopefully in the not-too-distant future.

## Figures and Tables

**Table 1:** Physico-chemical properties of unmodified CeO_2_, redox-modified CeO_2_ and TiO_2_ NPs. The hydrodynamic diameter of the MNMs was determined directly after preparing the suspensions of 125 µg/mL in water or in RPMI 1640 without FBS (0 h) and after 24 h incubation at 37 °C, 5% CO_2_. Values are means ± SD of three particle suspensions prepared independently. The effective density in the exposure medium was determined by the method described in [40].

	CeO_2_-A (Undoped)	CeO_2_-C (27% Zr)	CeO_2_-E (78% Zr)	TiO_2_^a^
Nominal diameter ^b^ [nm]	20	20	20	21
z-average in water [nm]	44.3 ^c^	58.1 ^c^	100 ^c^	165 ± 19 ^d^
z-average in RPMI-FBS, 0 h [nm]	3063 ± 437 ^c^ PDI: 0.257	6355 ± 590 ^c^ PDI: 0.347	7724 ± 322 ^c^ PDI: 0.389	1918 ± 309 ^e^ PDI: 0.263
z-average in RPMI-FBS, 24 h [nm]	4167 ± 178 ^c^ PDI: 0.206	6451 ± 935 ^c^ PDI: 0.477	8858 ± 1582 ^c^ PDI: 0.586	2903 ± 68 ^e^ PDI: 0.187
Material density [g/cm^3^]	7.22 ^f^	6.80 ^f^	6.02 ^f^	4.23 ^b^
Effective density in RPMI-FBS [g/cm^3^]	1.24 ^e^	1.12 ^e^	1.09 ^e^	1.32 ^e^

^a^ 80% anatase/20% rutile, ^b^ data from the particle provider, ^c^ determined by DLS with 1:10 diluted stock solution in water, related to number, ^d^ determined by DLS at 1 mg/mL related to intensity, ^e^ determined at 125 µg/mL, ^f^ data from [22].

**Table 2:** Characteristics of the CeO_2_ and TiO_2_ aerosols and estimated doses after ALI exposure.

	CeO_2_-A (Undoped)	CeO_2_-C (27% Zr)	CeO_2_-E (78% Zr)	TiO_2_
Modal value x_M_ [nm]c/6_g_	49/1.31	52/1.34	48/1.32	47/1.24
Total number concentration c_N_ [1/cm^3^]	1.75 × 10^5^	1.37 × 10^5^	2.07 × 10^5^	2.8 × 10^5^
Mass concentration c_MS_ [mg/m^3^] ^a^	1.77	1.66	2.24	2.2
Dose 0.5 h − EF [µg/cm^2^] ^b^				0.02–0.03
Dose 4 h − EF [µg/cm^2^] ^b^	0.19 ± 0.09	0.18 ± 0.02	0.24 ± 0.01	0.17
Dose 0.5 h + EF [µg/cm^2^] ^c^				0.15–0.18
Dose 4 h + EF [µg/cm^2^] ^c^	0.93 ± 0.44	0.88 ± 0.09	1.19 ± 0.07	1.14

^a^ calculated from SMPS with material density ρ (see Table 1), ^b^ without electrostatic field (EF), dose estimated on the basis of fluorescein deposition [12], ^c^ with EF (−1000 V), dose estimated on the basis of fluorescein deposition and enhancement factor due to the EF.

**Table 3:** LOAELs derived from several endpoints upon exposure to TiO_2_ NPs. As a metric for direct comparison the specific particle surface area (cm^2^) per average rat lung surface area (cm^2^) [45] or cultured cell surface area (cm^2^) is chosen.

Endpoints	Marker	Lung Exposure	ALI Exposure	Submerged Exposure
Cytotoxicity	LDH	0.15 ^a^	0.09 ^b^	41.2 ^b^
Genotoxicity	Comet Assay	0.03 ^c^	0.57 ^d^	5.7 ^d^
	DSBs	0.57 ^e^	0.09 ^f^	n.d.
Inflammation	IL-8	0.57 ^g^	0.09 ^h^	10.3 ^g^
	TNF-α	0.57 ^g^	0.57 ^h^	n.a.

^a,b^ release was analyzed by the LDH assay as described in [36] and Methods section, respectively; ^c^ measured by the alkaline comet assay as described in [36]; ^d^ determined by the fpg based and alkaline comet assay as described in Methods section; ^e^ DNA double strand breaks (DSBs) were analyzed with the γH2AX assay as described in [36] and ^f^ via the analysis of p53 binding protein 1 foci as described in the Supplementary Methods section; ^g^ release was measured by ELISA as described in [36] and in Methods section; ^h^ gene expression was measured by RT-qPCR as described in Methods section; n.d.: not detectable, n.a.: not analyzed.

## Data Availability

The data presented in this study are available on request from the corresponding authors.

## Supplementary Materials

The following are available online at https://www.mdpi.com/2079-4991/11/1/65/s1, Supplementary Methods, Table S1: Physico-chemical properties of TiO_2_ P25 (identical to NM-105), Table S2: Overview of particle doses, Table S3: Primers for RT-qPCR experiments, Figure S1: Aerosol generation from NP suspensions, Figure S2: The unmodified and redox-modified CeO_2_ NPs have no effect on viability of THP-1 macrophages but slightly increase IL-8 release, Figure S3: Cell-free DCFH oxidation by TiO_2_ NPs, Figure S4: No impact on target gene expression in A549 cells exposed to TiO_2_ NPs under submerged conditions in FBS-free medium for 4 h, Figure S5: TiO_2_ NPs increase 53BP1 foci formation in A549 cells at the ALI in accordance with induced strand breaks and alkali-labile sites as shown in Figure 6.
