# Supplementary material for: Air–Liquid Interface Exposure of Lung Epithelial Cells to Low Doses of Nanoparticles to Assess Pulmonary Adverse Effects
Source: Nanomaterials (Basel). 2020 Dec 29;11(1):65. doi: 10.3390/nano11010065 (PMC7823463; doi:10.3390/nano11010065)
Supplement: Supplementary file 1 [file nanomaterials-11-00065-s001.pdf]

Supplementary data

Air–Liquid Interface Exposure of Lung Epithelial Cells to Low Doses of Nanoparticles to Assess Pulmonary Adverse Effects

Silvia Diabaté [1,*,†], Lucie Armand [2,†], Sivakumar Murugadoss [1], Marco Dilger [1], Susanne Fritsch-Decker [1], Christoph Schlager [3], David Béal [2], Marie-Edith Arnal [2], Mathilde Biola-Clier [2], Selina Ambrose [4], Sonja Mülhopt [3], Hanns-Rudolf Paur [3], Iseult Lynch [5], Eugenia Valsami-Jones [5], Marie Carrière [2,†] and Carsten Weiss [1,*,†]

1 Karlsruhe Institute of Technology, Institute of Biological and Chemical Systems–Biological Information Processing, 76344 Eggenstein-Leopoldshafen, Germany; sivakumar.murugadoss@kuleuven.be (S.-K.M.); marcodilger@gmx.de (M.D.); susanne.fritsch-decker@kit.edu (S.F.-D.)
2 CEA, CNRS, IRIG, SyMMES, University Grenoble Alpes, 38054 Grenoble, France; lucie.armand@agroparistech.fr (L.A.); david.beal@cea.fr (D.B.); marie-edith.arnal@wanadoo.fr (M.-E.A.); mathilde.clier@gmail.com (M.B.-C.); marie.carriere@cea.fr (M.C.)
3 Karlsruhe Institute of Technology, Institute for Technical Chemistry, 76344 Eggenstein-Leopoldshafen, Germany; c.schlager@vitrocell.com (C.S.); sonja.muelhopt@kit.edu (S.M.); hanns@dr-paur.net (H.-R.P.)
4 Promethean Particles Ltd., Nottingham, NG7 3EF, UK; selina.ambrose@proparticles.co.uk
5 School of Geography Earth & Environmental Sciences (GEES), University of Birmingham (UoB), Edgbaston, Birmingham, B15 2TT, UK; i.lynch@bham.ac.uk (I.L.); E.ValsamiJones@bham.ac.uk (E.V.-J.)
* Correspondence: silvia.diabate@kit.edu (S.D.); carsten.weiss@kit.edu (C.W.); Tel.: +49-72160822692 (S.D.); +49-72160824906 (C.W.)
† Equal first and last author contribution.

Supplementary Methods

Cell-free DCF test

The test uses the oxidation of the non-fluorescent 2′,7′-dichlorodihydrofluorescein ($DCFH_2$) to the fluorescent 2′,7′-dichlorofluorescein (DCF) as an indicator for the presence of reactive oxygen species [1-2] and has been performed as described previously [3]. Briefly, $DCFH_2$-diacetate was deacetylated with NaOH by mixing 0.1 mL of 5 mM $DCFH_2$-DA (Invitrogen, Karlsruhe, Germany) in ethanol with 2.4 mL of 0.01 N NaOH and incubating at room temperature (24°C) for 30 min. For neutralization, 10 mL PBS was added and kept on ice in the dark until use. Just prior to use, horseradish peroxidase (HRP, Sigma, Taufkirchen, Germany) was added as a catalyst (2.2 U/mL). The $DCFH_2$ concentration in the working solution was 40 µM.

Suspensions of test particles were prepared in PBS (10 mg/mL), sonicated for 10 min and further diluted, and H_2O_2 standard preparations (0.04 to 10 μM) were also prepared. The test solutions were mixed 1:1 (v/v) with the prepared $DCFH_2$ solution and incubated at 37°C for 15 min in the dark. Then, solutions were centrifuged (20,000 × g for 15 min) to remove the particles and the fluorescence of the supernatant was monitored at 485 nm excitation and 530 nm emission using a fluorescence microplate reader (BIO-TEK FL600 from MWG-Biotech AG, Ebersberg, Germany). Results were expressed as fold changes relative to the particle free sample.

Culture of THP-1 cells and differentiation to macrophages

The human myeloid leukemia cell line THP-1 was obtained from DSMZ (German Collection of Microorganisms and Cell Cultures, Braunschweig, ACC 16). Cells were cultured in RPMI 1640 medium containing 10 % FBS, 2 mM L-glutamine, 100 µg/mL penicillin, and 100 U/mL streptomycin. The non-adherent THP-1 monocytes were differentiated into macrophage-like cells by treatment with 30 ng/mL TPA (12-O-tetradecanoylphorbol-13-acetate, Sigma, Taufkirchen) for 4 days and incubation in TPA-free medium for 3 days [4]. TPA-differentiated THP-1 cells (dTHP-1) resemble some biological and morphological characteristics of human alveolar macrophages, such as eicosanoid and cytokine production [5]. The dTHP-1 cells become adherent and no longer divide. The differentiation was confirmed by detection of CD14 expression using flow cytometry.

MCP-1 release

Secreted monocyte chemotactic protein-1 (MCP-1) was analyzed in the cell culture medium using the MCP-1 ELISA kit from eBioscience (Frankfurt, Germany) according to the manufacturer's instructions. For measurement of absorption and data analysis, a microplate reader and the software package SoftMaxPro (Molecular Devices, Ismaning, Germany) were used.

Gene expression and genotoxicity studies under submerged conditions

TiO_2 NPs were pre-wetted with ethanol and suspended in 0.5 mg/mL sterile BSA at the concentration of 5 mg TiO_2/mL according to the NanoGenoTox protocol [6]. Immediately before cell exposure, they were sonicated for 15 min at 70% of amplitude, at 4°C, using an indirect cup-type sonicator (cup-horn), operated via a Vibracell 75041 sonicator (Fisher Bioblock, Rungis, France). They were then diluted in cell culture medium (DMEM without FBS) and applied to cells.

Analysis of p53 binding protein 1 (53BP1) foci

DNA double strand breaks (DSB) or replication fork blockade were determined by counting p53 binding protein 1 (53BP1) foci in cell nuclei. 53BP1 is a non-enzymatic protein which is recruited shortly after primary DSB detection. This protein is homogeneously distributed in the nuclei of unperturbed cells and it is recruited within 1-2 min to DSB sites [7]. Like gamma-H2AX it can therefore serve as a marker for DSB. The method has been performed as described previously [8]. As a positive control, cells were exposed to 25 µM etoposide (Sigma-Aldrich) for 24 h. Briefly, cells were fixed for 20 min in 3% paraformaldehyde (Sigma-Aldrich), stained using anti-53BP1 antibody (Novus Biologicals, Littleton, CO, USA, 1/500 vol./vol.) and slides were mounted with Fluoroshield (Sigma-Aldrich) containing DAPI (1 slide per condition). 53BP1 foci were visualized on an Axio ImageA1 microscope coupled to an Axiocam MRm camera (Carl Zeiss). At least 15 images per condition were captured; on each image both, total number of 53BP1 foci and total number of nuclei, were determined. Apoptotic cells and dividing cells were rejected.

Table S1. Physico-chemical properties of TiO_2 P25 (identical to NM-105) from [9].

Chemical composition [a]	99.8 % (w/w) TiO_2
Coating	no
Solubility [b]	1 %
z-average in HNO_3 10^{-2} M [c]	128 nm by intensity
Material density	4.23 g/cm³
Specific surface area [d]	50 ± 15 m²/g [e]<br>46.18 – 52.81 m²/g [f]
Crystallinity	80 : 20 (anatase : rutile) [g]
Size of primary particles	21 – 24 nm [h]

[a] determined by energy-dispersive X-ray spectroscopy (EDS), [b] tested at 0.32 mg/mL after 24 h incubation in Caco2 medium, [c] TiO_2 NP suspensions dispersed by sonication, [d] determined by the Brunauer, Emmett and Teller (BET) method [10], [e] BET determined by the producer, [f] BET determined by different laboratories [9], [g] determined by different laboratories [9], [h] determined by TEM by different laboratories [9].

Table S2. Overview of particle doses under submerged conditions and 24 h exposure (a) as well as under air-liquid interface and submerged conditions for the indicated exposure times (b). For convenient comparison to the literature, different metrics were selected i.e. mass of NPs/volume of cell culture medium (µg/mL), mass of NPs/ cellular surface area (µg/cm²) and surface area of NPs/ cellular surface area (cm²/cm²). In case of titania NPs, surface area was provided by the manufacturer (see Table S1) whereas for ceria NPs surface area was calculated based on the diameter as listed in Table 1.

(a)

	CeO_2-A			CeO_2-C			CeO_2-E			TiO_2						
µg/mL	31.3	62.5	125	31.3	62.5	125	31.3	62.5	125	0.5	3	15.6	31.3	62.5	125	250
µg/cm^2	10.3	20.6	41.2	10.3	20.6	41.2	10.3	20.6	41.2	0.17	1.14	5.2	10.3	20.6	41.2	82.4
cm^2/cm^2	4.28	8.56	17.12	4.54	9.09	18.18	5.13	10.27	20.53	0.09	0.57	2.6	5.15	10.3	20.6	41.2

(b)

	Air-Liquid Interface Exposure									Submerged Exposure	
	CeO_2-A		CeO_2-C		CeO_2-E		TiO_2			TiO_2	
	4 h		4 h		4 h		0.5 h	4 h		4 h	
µg/mL	n.a.	n.a.	n.a.	n.a.	n.a.	n.a.	n.a.	n.a.		0.5	3
µg/cm^2	0.19	0.93	0.18	0.88	0.24	1.19	0.17	0.17	1.14	0.17	1.14
cm^2/cm^2	0.08	0.39	0.08	0.39	0.12	0.59	0.09	0.09	0.57	0.09	0.57

n.a. = not applicable

Table S3. Primers for RT-qPCR experiments.

	**Forward**	**Reverse**
HO-1	TTCTCCGATGGGTCCTTACACT	GGCATAAAGCCCTACAGCAACT
IL-1β	ACAGATGAAGTGCTCCTTCCA	GTCGGAGATTCGTAGCTGGAT
IL-8	GAATGGGTTTGCTAGAATGTGATA	CAGACTAGGGTTGCCAGATTTAAC
MCP-1	CATTGTGGCCAAGGAGATCTG3	TTCGTTTCCCTTTGAGGCTTC
TNF-α	GAGCAGTGAAAGCATGATCC	CGAGAAGATGATCTGACTGCC
GAPDH	GAGTCAACGGATTTGGTCGT	TTGATTTTGGAGGGATCTCG
S18	AACGTCTGCCCTATCAACTTT	TGGATGTGGTAGCCGTTTCT
CycloA	TTCATCTGAACTGCCAAGAC	TTGATTTTGGAGGGATCTCG

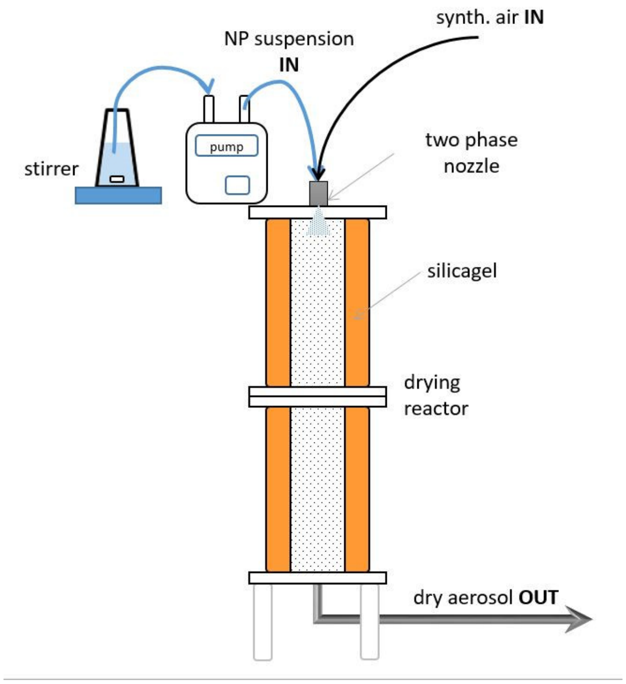

Figure S1. Aerosol generation from NP suspensions. The continuously stirred suspension of NPs is dispersed via a two-phase nozzle into the aerosol reactor. Inside the reactor the aerosol is dried and is led to the automated exposure station.

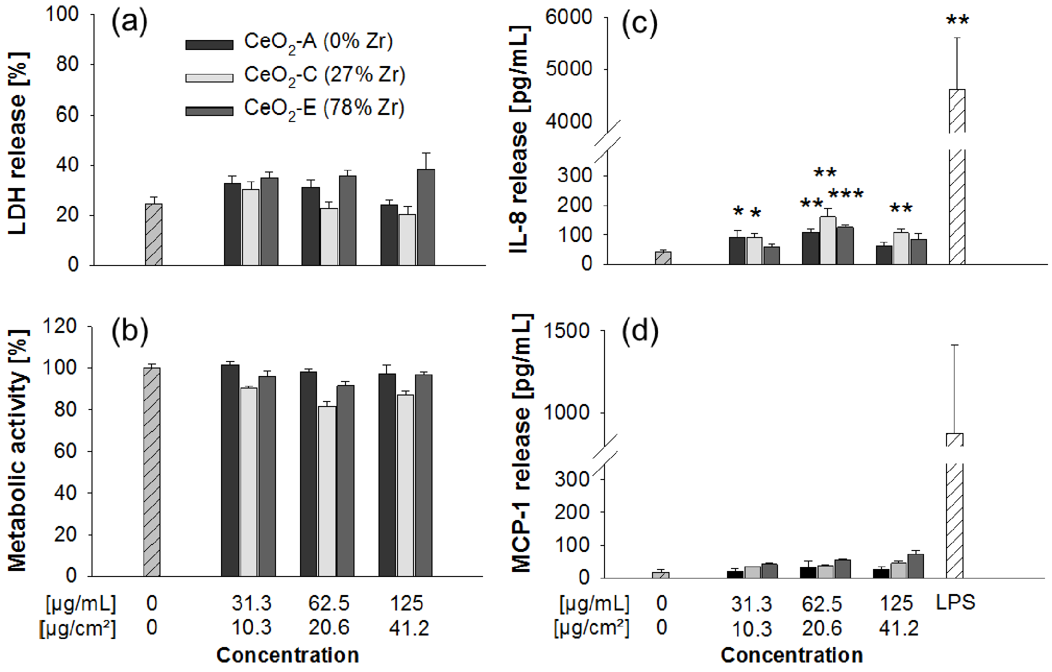

Figure S2. The unmodified and redox-modified CeO_2 NPs have no effect on viability of THP-1 macrophages but slightly increase IL-8 release. The cells were seeded as mono-culture and exposed to CeO_2-NPs in medium without serum for 24h. LDH release **(a),** AlamarBlue assay **(b),** IL-8 **(c)** and MCP-1 release **(d)** were performed as described in Figure S2. The positive control was exposed to 0.1 µg/mL LPS. The results are means ± s.e.m. of two independent experiments performed in duplicate. *p < 0.05, **p < 0.01 and ***p < 0.001 indicate significant differences of treated cells compared to control cells exposed to medium only.

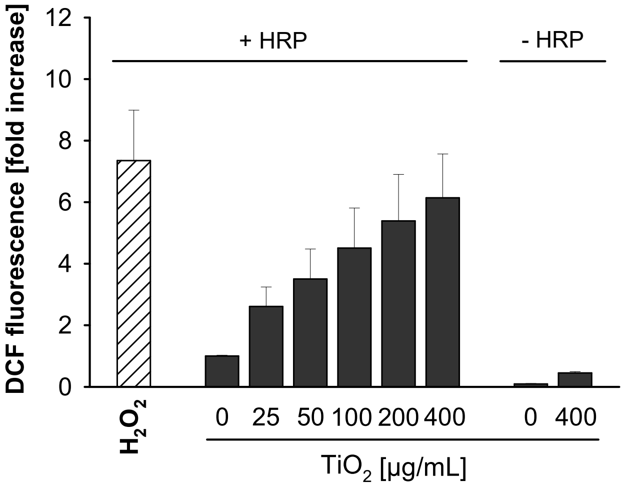

Figure S3. Cell-free DCFH oxidation by TiO_2 NPs. The NPs were tested by the cell-free DCF test and 0.3 µM H_2O_2 was used as the positive control. Fluorescence units were normalised to a control solution without NPs. The highest NP concentration as well as the control solution without NPs were additionally tested with the reagents without horse radish peroxidase (-HRP). The data represent mean values ± s.e.m of two experiments with three replicates.

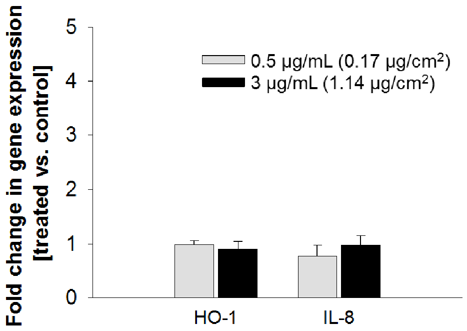

Figure S4. No impact on target gene expression in A549 cells exposed to TiO_2 NPs under submerged conditions in FBS-free medium for 4 h. The deposited doses were adjusted to those calculated in the ALI exposure experiments (0.17 and 1.14 µg/cm²). The data represent mean values from three independent exposure experiments ± SD.

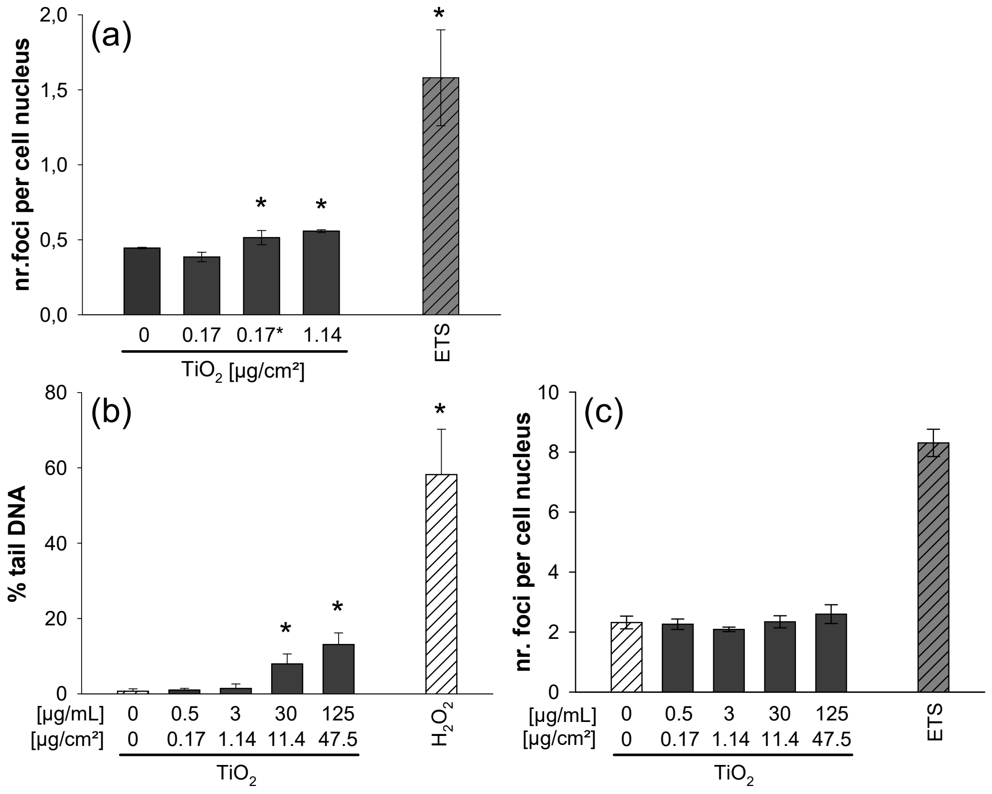

Figure S5. TiO_2 NPs increase 53BP1 foci formation in A549 cells at the ALI in accordance with induced strand breaks and alkali-labile sites as shown in Figure 6. However, under submerged conditions strand breaks are only provoked at much higher doses yet no enhanced 53BP1 foci formation could be observed. **(a)** At the ALI, the cells were exposed to clean air or to TiO_2-NPs at the indicated doses but for different time periods (0.17 µg/cm²: 30 min aerosol, 0.17* µg/cm²: 30 min aerosol + 3 h 30 min air, 1.14 µg/cm²: 4 h aerosol) and 53BP1 foci were monitored. Cells were also exposed under submerged conditions **(b, c)** and the alkaline comet assay was performed (positive control: 50 µM H_2O_2) **(b),** or 53BP1 foci were analyzed (positive control: 50 µM etoposide) **(c).** Data are means of three independent experiments ± SD. Statistics *$p < 0.05$, exposed vs. control (clean air (ALI) or unexposed cells (submerged)).

1. References

1. Cathcart, R.; Schwiers, E., Ames, B.N. Detection of picomole levels of hydroperoxides using a fluorescent dichlorofluorescein assay. *Anal. Biochem* **1983**, 134, 111-116.
2. Foucaud, L.; Wilson, M.R.; Brown, D.M., Stone, V. Measurement of reactive species production by nanoparticles prepared in biologically relevant media. *Toxicol. Lett* **2007**, 174, 1-9.
3. Mülhopt, S.; Diabaté, S.; Dilger, M.; Adelhelm, C.; Anderlohr, C.; Bergfeldt, T.; Gomez, d.l.T.; Jiang, Y.; Valsami-Jones, E.; Langevin, D.; et al. Characterization of Nanoparticle Batch-To-Batch Variability. *Nanomaterials (Basel)* **2018**, 8, 311.
4. Braakhuis, H.M.; Giannakou, C.; Peijnenburg, W.J.; Vermeulen, J.; van, L.H., Park, M.V. Simple in vitro models can predict pulmonary toxicity of silver nanoparticles. *Nanotoxicology* **2016**, 10, 770-779.
5. Chen, F.; Kuhn, D.C.; Gaydos, L.J., Demers, L.M. Induction of nitric oxide and nitric oxide synthase mRNA by silica and lipopolysaccharide in PMA-primed THP-1 cells. *APMIS* **1996**, 104, 176-182.
6. Jensen, K.A.; Kembouche, Y.; Christiansen, E.; Jacobsen, N.R.; Wallin, H.; Guiot, C.; Spalla, O., Witschger, O. Final protocol for producing suitable manufactured nanomaterial exposure media - Standard Operation Procedure (SPO) and background documentation. 2011, Available online: https://www.anses.fr/en/system/files/nanogenotox_deliverable_5.pdf (Accessed on 04.08.2020).
7. Bekker-Jensen, S., Mailand, N. Assembly and function of DNA double-strand break repair foci in mammalian cells. *DNA Repair (Amst)* **2010**, 9, 1219-1228.
8. Armand, L.; Tarantini, A.; Beal, D.; Biola-Clier, M.; Bobyk, L.; Sorieul, S.; Pernet-Gallay, K.; Marie-Desvergne, C.; Lynch, I.; Herlin-Boime, N.; et al. Long-term exposure of A549 cells to titanium dioxide nanoparticles induces DNA damage and sensitizes cells towards genotoxic agents. *Nanotoxicology* **2016**, 10, 913-923.
9. Rasmussen, K.; Mast, J.; De Temmerman, P.J.; Verleysen, E.; Waegeneers, N.; Van Steen, F.; Pizzolon, J.C.; De Temmerman, L.; Van Doren, E.; Jensen, K.A.; et al. *Titanium dioxide, NM-100, NM-101, NM-102, NM-103, NM-104, NM-105: Characterisation and physico-chemical properties*, European Union, Luxembourg: Publications Office of the European Union, 2014; pp. 1-218.
10. Brunauer, S.; Emmett, P.H., Teller, E. Adsorption of gases in multimolecular layers. *J. Am. Chem. Soc* **1938**, 60, 309-319.
